# Prognosis and metabolism with a Golgi apparatus-related genes-based formula in breast cancer

**DOI:** 10.1097/MD.0000000000039177

**Published:** 2024-08-16

**Authors:** Hang Lu, Xin Yu, Wenge Li, Yimin Zhang, Shengrong Sun

**Affiliations:** a Department of Breast and Thyroid Surgery, Renmin Hospital of Wuhan University, Wuhan, China; b Department of Cardiovascular Surgery, Xijing Hospital, Xi’an, China; c Department of Oncology, Shanghai Artemed Hospital, Shanghai, China.

**Keywords:** breast cancer, Golgi apparatus, immune, metabolism, mutation, prognosis

## Abstract

The Golgi apparatus (GA), an organelle that processes, sorts, and transports proteins synthesized by the endoplasmic reticulum, is also involved in many cellular processes associated with cancer, such as angiogenesis, the innate immune response, and tumor invasion and migration. We aimed to construct a breast cancer (BC) prognosis prediction model based on GA-related genetic information to evaluate the prognosis of patients with BC more accurately than existing models and to stratify patients for clinical therapy. In this study, The Cancer Genome Atlas-breast invasive carcinoma was used as the training cohort, and the Molecular Taxonomy of Breast Cancer International Consortium cohort was used as the validation cohort. Using bioinformatics methods, we constructed a GA-related gene risk score (GRS). The GRS was used to divide BC patients into a high-GRS group and a low-GRS group, and functional analysis, survival analysis, mutation analysis, immune landscape analysis, and metabolic analysis were performed to compare the 2 groups. Finally, a nomogram was constructed for clinical application. The genes in the GRS model were mainly related to the glucose metabolism pathway, and the main mutations in the 2 groups of patients were mutations in TP53 and CHD1. The mutation rate in the high-GRS group was greater than that in the low-GRS group. The high GRS group had higher tumor immune activity glycolysis; the pentose phosphate pathway tended to be the dominant metabolic pathways in this group, while fatty acid oxidation and glutamine catabolism tended to be dominant in the low-GRS group. GA-related genes were used to construct a prediction model for BC patients and had high accuracy in predicting prognosis. The mutations associated with the GRS are mainly TP53 and CDH1. Interestingly, the GRS is correlated with glucose metabolism in terms of gene expression and functional enrichment. In summary, the role of GRS-related genes in glucose metabolism is worthy of further study.

## 1. Introduction

Breast cancer (BC) ranks first among malignant tumors in terms of incidence in females, and breast invasive carcinoma (BRCA) is the main type.^[1,2]^ The classic molecular subtype classification of BC is mainly applied to guide the selection of individualized therapy for patients, but owing to the high heterogeneity of breast carcinoma, classical protocols are not sufficient to precisely predict the prognosis of patients with breast carcinoma. Thus, it is necessary to establish a systematic and accurate approach to predict the prognosis of BC.

The Golgi apparatus (GA) is an organelle that processes, sorts, and transports proteins synthesized by the endoplasmic reticulum and then sorts them into specific parts of the cell or secretes them out of the cell, such as through the glycosylation of proteins.^[3]^ The GA is also involved in many cellular processes associated with cancer, including angiogenesis, the innate immune response, and tumor invasion and migration.^[4–7]^ Therefore, the GA has been targeted for tumor therapy.^[6,8–12]^

Abnormal glycosylation on the surface of BC cells and the overexpression of some Golgi proteins, such as Golgi phosphorylated protein 3, are associated with poor outcomes and a poor prognosis in BC patients.^[13,14]^ A recent study revealed that mechanistic target of rapamycin complex 1 controls the GA structure and vesicle secretion by phosphorylating SCYL1.^[15]^ Abnormal N-glycan remodeling and metabolism in the GA are associated with epithelial-mesenchymal transformation and metastasis in breast cancer patients.^[16]^ Therefore, the expression of GA-related genes has the potential to predict the prognosis of breast cancer patients. Recently, genetic algorithms related to GA gene pairs and cancer have also attracted increased amounts of attention. For example, Jiang et al^[17]^ constructed a GA-associated gene set formula to predict the prognosis of lung adenocarcinoma patients and obtained relatively accurate outcomes. In this study, we aimed to use bioinformatics to construct a BC prognosis prediction system based on GA-related gene expression to evaluate the prognosis of BC with better accuracy than existing models and to stratify patients to provide a reference for the clinical treatment of BC.

## 2. Materials and methods

### 2.1. Data acquisition

The clinical survival, gene expression, and mutation data of BRCA patients were obtained from The Cancer Genome Atlas (TCGA) database^[18]^ and the Molecular Taxonomy of Breast Cancer International Consortium (METABRIC) database.^[19]^ The genomic and clinical data of BRCA patients were obtained from the Cancer Genome Browser (http://xena.ucsc.edu/) of the University of California at Santa Cruz. The genomic and clinical information of the METABRIC cohort was obtained from a platform dedicated to multi-omics tumor genetic analysis (https://www.cbioportal.org/study/summary?id=brca_metabric). The TCGA-BRCA dataset was used as the training cohort, while data from the METABRIC database were used as the validation cohort. We collected 1091 breast cancer samples and 113 normal tissue samples from the TCGA database, and there were 1904 breast cancer samples from the METABRIC database. The RNA-seq data of the TCGA database in fragments per kilobase of exon per million mapped fragments values and matched medical data were retrieved from the University of California at Santa Cruz Xema data portal. Subsequently, the fragments per kilobase of exon per million mapped fragments values were converted into transcripts per kilobase million values. The METABRIC data were standardized according to the Z-score. A total of 1613 GA-related genes were extracted from the Molecular Signatures Database.

### 2.2. Golgi-related gene stratification

The TCGA-BRCA database contains information on the expression of GA-related genes, which we collected for quantification. According to Fc (fold change) <2/3 or >3/2 and *P* < .05, the Wilcoxon test was used to identify differentially expressed genes (DEGs) between BRCA tissues and normal mammary tissues. Genes with Fc >3/2 and Fc <2/3 were considered to be upregulated and downregulated, respectively. DEGs are shown in the heatmap.

### 2.3. Functional analysis of the DEGs

The R software package “clusterprofiler” (https://git.bioconductor.org/packages/clusterProfiler) was used for Kyoto Encyclopedia of Genes and Genomes (KEGG) and gene ontology (GO) pathway enrichment analysis. The corrected *P* value threshold for the error discovery rate was set to .05 with the use of Fisher exact test.

### 2.4. Derivation and verification of the risk score model based on GA-related genes

First, we cross-checked the RNA expression in the TCGA-BRCA and METABRIC datasets to identify differentially and coexpressed expressed GA-related genes. Then, univariate Cox analysis was used to select the GA-related genes associated with prognosis. Then, LASSO Cox regression analysis was performed to shrink and select variables using the “glmnet” R package. Ten-fold cross-validation was used to determine the optimal values of the penalty parameter λ in the LASSO Cox regression model. Subsequently, we used multivariate Cox regression analysis for further screening. Finally, we constructed the GA-related gene risk score (GRS) with the best possible genes.

The GRS was computed based on the following formula: risk score = Σ genes Cox quotient × genes expression. We used the R package “surv_cutpoint” in “survminer” with the median expression value as the cutoff, the lower-value group was defined as the high-risk group, and the higher-value group was defined as the low-risk group. The R package “survivalROC” was used to estimate the predictive sensitivity of the GRS.^[20]^ To verify the validity of the model, the correlation coefficients and cutoff values used in the validation cohort were the same as those used in the training cohort.

### 2.5. Immune landscape analysis

CIBERSORT is a robust analytic tool based on the gene expression profiles of 547 genes that can be used to accurately quantify different tumor-infiltrating immune cells. The immune infiltration data were downloaded to determine the abundances of 22 immune cell types using the CIBERSORT algorithm.^[21]^ Correlations were evaluated by Spearman correlation analysis.

### 2.6. Construction of the nomogram

In this article, we constructed an OS prediction nomogram with 1-, 3-, and 5-year OS as endpoints using the Cox regression model along with the R package “rms.” The discriminative ability of the nomogram was assessed by using the C-index. Calibration plots were used to evaluate the consistency between the actual and predicted OS data.

### 2.7. Statistical methods

The Wilcoxon test was used to screen the DEGs. The prognostic value of the related genes was determined by univariate and multivariate Cox analyses of OS. The logarithmic rank test was performed to compare the Kaplan–Meier survival curves of the 2 groups. R version 4.0.0 (R-project.org) was used to perform all the statistical analyses. *P* ≤ .05 was considered to indicate statistical significance. The study was reviewed and approved by The Ethics Committee of Renmin Hospital of Wuhan University.

## 3. Results

**Figure 1.**
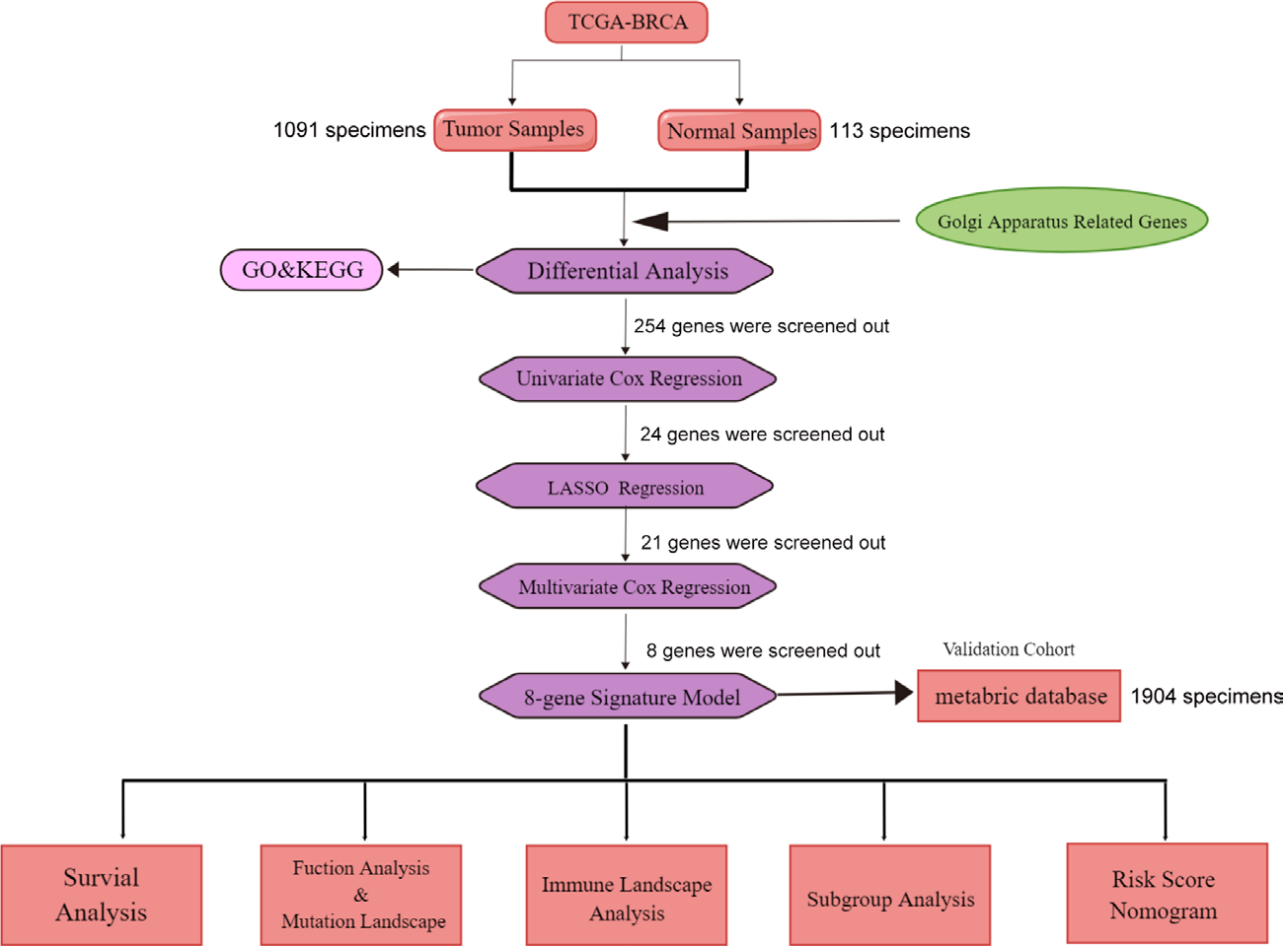
Flowchart of the study. BRCA = breast invasive carcinoma, GO = gene ontology, KEGG = Kyoto Encyclopedia of Genes and Genomes, LASSO = least absolute shrinkage and selection operator.

We selected 1091 and 1904 BRCA patients from the training sets and the validation sets, respectively. Figure 1 shows the flowchart of this study. The clinical baseline data of these patients are summarized in Table 1.

**Table 1:** Clinical information of patients.

Database	TCGA	Metabric
Number of patients	n = 1091	n = 1904
Age		
<60	579 (53.12%)	842 (44.22%)
≥60	511 (46.88%)	1062 (55.78%)
PAM50		
Normal	40 (3.67%)	54 (2.84%)
Lum A	564 (51.70%)	598 (31.41%)
Lum B	215 (19.71%)	764 (40.13%)
Basal	190 (17.42%)	244 (12.82%)
HER2	82 (7.52%)	244 (12.82%)
Stage		
I/II	800 (74.98%)	1274 (91.13%)
III/IV	267 (25.02%)	124 (8.87%)

TCGA = The Cancer Genome Atlas.

### 3.1. Differential expression of Golgi apparatus-related genes

**Figure 2.**
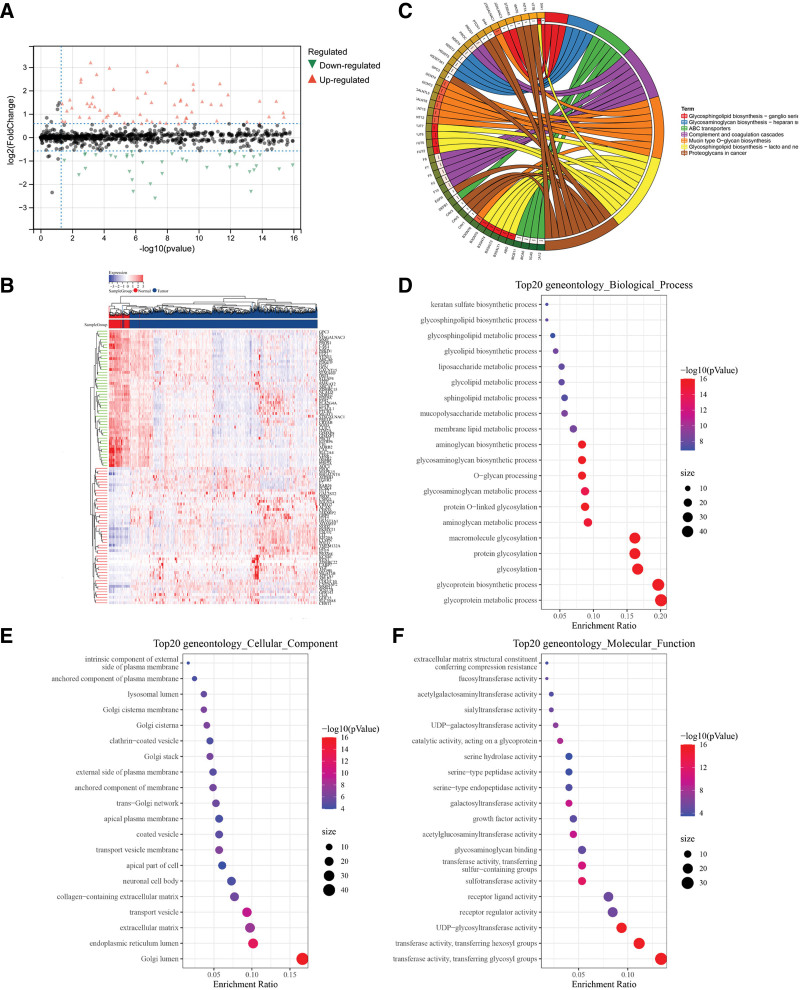
Screening of differentially expressed Golgi apparatus-related genes. (A) Volcanic map of differential expression of Golgi apparatus-related genes. (B) Heat maps of differentially expressed Golgi apparatus-related mRNAs. (C) Chord diagram of KEGG pathway of Golgi associated DEGs. (D) Biological process of GO enrichment of Golgi apparatus-related DEGs. (E) Cellular component of GO enrichment of Golgi apparatus-related DEGs. (F) Molecular function of GO enrichment of Golgi apparatus-related DEGs. DEGs = differentially expressed genes, GO = Gene Ontology, KEGG = Kyoto Encyclopedia of Genes and Genomes.

The Wilcoxon test was performed for the DEGs in normal breast tissues and BRCA tissues identified according to the parameter of Fc <2/3 or >3/2. We identified 254/1610 GA-related DEGs (Supplementary Table S1, Supplemental Digital Content, http://links.lww.com/MD/N356). DEGs with Fc <2/3 and >3/2 represented downregulated and upregulated genes, respectively (Fig. 2A). We identified 116 upregulated genes and 138 downregulated genes (Fig. 2B).

### 3.2. Functional analysis of GA-related DEGs

We performed KEGG analysis of GA-related genes and identified significantly enriched KEGG pathways. These categories were mainly related to glycosphingolipid and glycosaminoglycan biosynthesis (Fig. 2C). Subsequently, we identified the most obviously significantly enriched category of GA-related genes through GO enrichment analysis. The most significant enriched GA-related DEGs were related to glycoprotein metabolism in the biological process category, Golgi lumen in the cellular component category, and transferase activity and transferring glycosyl groups in the molecular function category (Fig. 2D–F).

### 3.3. Construction and evaluation of the predictive ability of the GRS model

**Figure 3.**
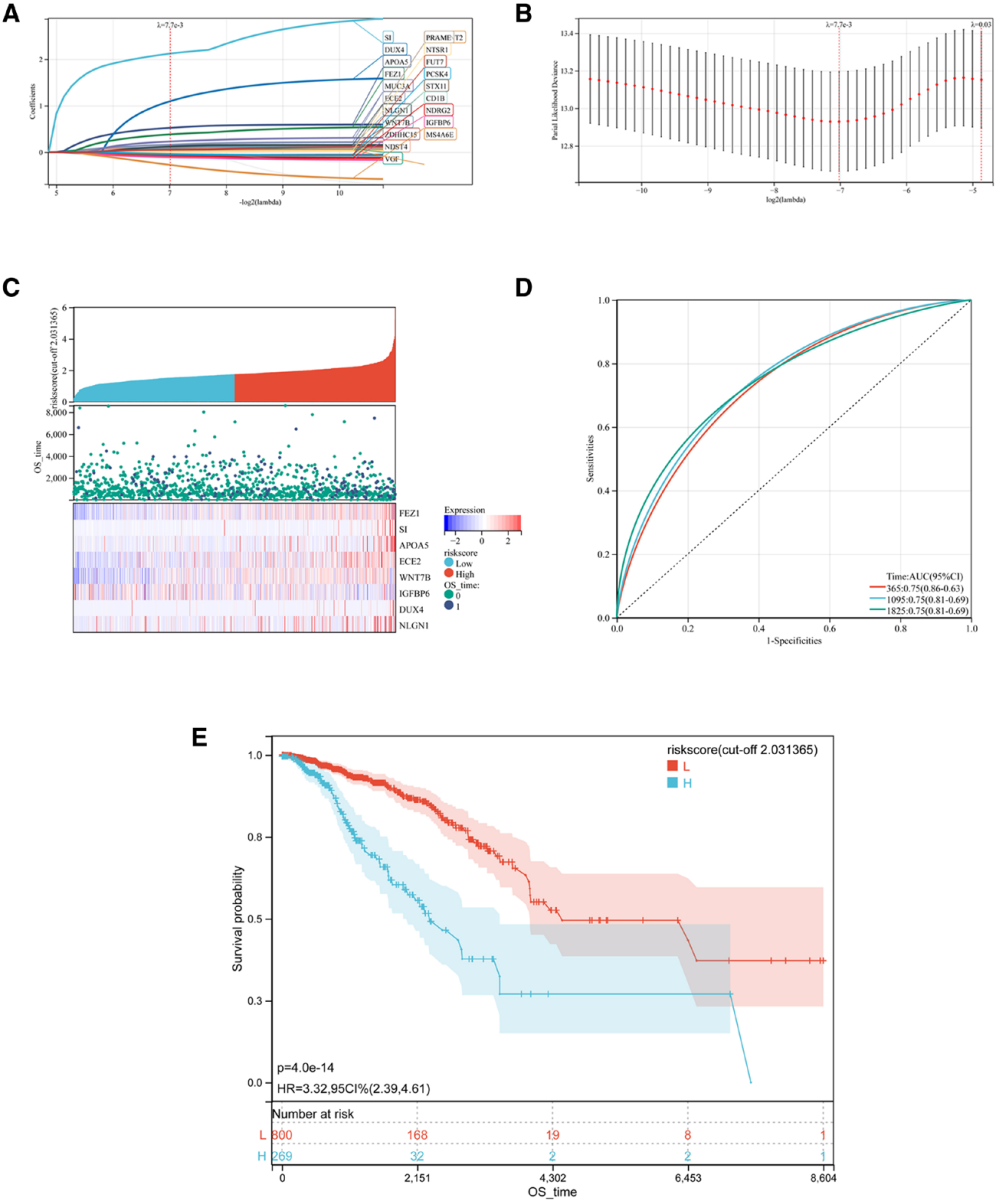
Construction of the GRS formula. (A) LASSO coefficient profile. (B) Penalty plot for the LASSO model with error bars. (C) The distribution, survival time, and survival state patterns of GRS in the training set, and heat maps of 8 prognostic genes for each patient. (D) Time-related ROC analysis in the training set. (E) Kaplan–Meier survival curve of the patients for OS in the training set. AUC = area under the curve, CI = confidence interval, GRS = Golgi apparatus gene-related risk score, HR = hazard ratio, LASSO = least absolute shrinkage and selection operator, OS = overall survival, ROC = receiver operating characteristic.

Twenty-four prognostic genes were identified from the 254 GA-related DEGs using univariate Cox regression analysis (Supplementary Figure S1, Supplemental Digital Content, http://links.lww.com/MD/N356). LASSO regression analysis was then performed to avoid overfitting of the prognostic model (Fig. 3A, B). After 21 genes were identified as potential candidates by LASSO regression analysis, multivariate Cox regression analysis was used for further screening. (Supplement Figure S2, Supplemental Digital Content, http://links.lww.com/MD/N356). Finally, 8 genes were identified as prognostic genes and used to develop the following risk score formula: GRS = FEZ1 × 0.532 + SI × 2.351 + APOA5 × 0.690 + ECE2 × 0.320 + WNT7B × 0.149 − IGFBP6 × 0.203 + DUX4 × 1.442 + NLGN1 × 0.291. Based on the median value, we divided 1091 patients into a high-GRS group and a low-GRS group (Fig. 3C). The OS of patients in the low-GRS subgroup was better than that of patients in the high-GRS group, as shown by Kaplan–Meier survival analysis (Fig. 3D) (hazard ratio = 3.32; 95% confidence interval [CI] = 2.39–4.61; *P* < .001). The area under the curves (AUCs) (95% CI) for predicting OS were 0.75 (0.86–0.63), 0.75 (0.81–0.69), and 0.75 (0.81–0.69) for 1-, 3- and 5-year OS, respectively (Fig. 3E). The risk model had a high accuracy in predicting OS in BRCA patients.

### 3.4. Verifying the stability of the GRS formula

**Figure 4.**
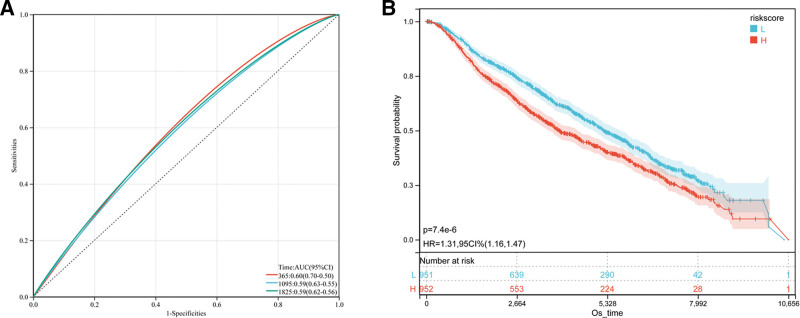
Verify the stability of GRS using validation sets. (A) The relationship between GRS and prognosis in the METABRIC was confirmed by time-related ROC analysis. (B) Kaplan–Meier survival curve of the patients in the high- and low-GRS groups for OS in the METABRIC. AUC = area under the curve, CI = confidence interval, GRS = Golgi apparatus gene-related risk score, HR = hazard ratio, METABRIC = Molecular Taxonomy of Breast Cancer International Consortium, OS = overall survival, ROC = receiver operating characteristic.

To confirm the stability of the GRS, we divided the patients in the METABRIC dataset into low and high GAR groups using the same threshold and risk formulas as in the training cohort. The AUC of the Kaplan–Meier curve showed that patients in the low-GRS subgroup had significantly longer OS than did those in the high-GRS subgroup (Fig. 4A) (hazard ratio = 1.31; 95% CI = 1.16–1.47; *P* < .001). The AUCs (95% CI) for OS at 1, 3, and 5 years were 0.60 (0.70–0.50), 0.59 (0.63–0.55), and 0.59 (0.62–0.55), respectively (Fig. 4B).

### 3.5. Functional analysis of the GRS groups

The Wilcoxon test was used to identify DEGs between the high- and low-GRS groups. The most significantly altered DEGs are listed in Supplementary Figure S3, Supplemental Digital Content, http://links.lww.com/MD/N356. KEGG analysis revealed that neuroactive ligand-receptor interaction was the pathway that was most differentially enriched between the high- and low-GRS groups (Supplementary Figure S4A, Supplemental Digital Content, http://links.lww.com/MD/N356). GO analysis revealed that skin development, presynapse, and cation transmembrane transporter activity were the most important pathways (Supplementary Figure S4B–D, Supplemental Digital Content, http://links.lww.com/MD/N356).

### 3.6. Identification of the GRS mutation landscape

Comparison of the mutant profiles of the 2 GRS groups indicated that the samples with higher GRSs contained more mutation events, and TP53 and CDH1 mutations were the most common mutations in these specimens (Supplementary Figure S4E, Supplemental Digital Content, http://links.lww.com/MD/N356).

### 3.7. Correlation between the GRS and immune status

The results of the association of the GRS with the immune features of BRCA patients showed that the proportions of M0 macrophages and activated dendritic cells were positively correlated with the GRS. The proportions of CD8 + T cells, naive B cells, activated NK cells, monocytes, and resting mast cells were negatively correlated with the GRS (Supplementary Figure S5, Supplemental Digital Content, http://links.lww.com/MD/N356). Although there was a mathematical correlation between the GRS and immune cell marker expression, the correlation coefficients were all low, which indicated a weak correlation overall.

### 3.8. Subgroup analysis with the GRS formula

**Figure 5.**
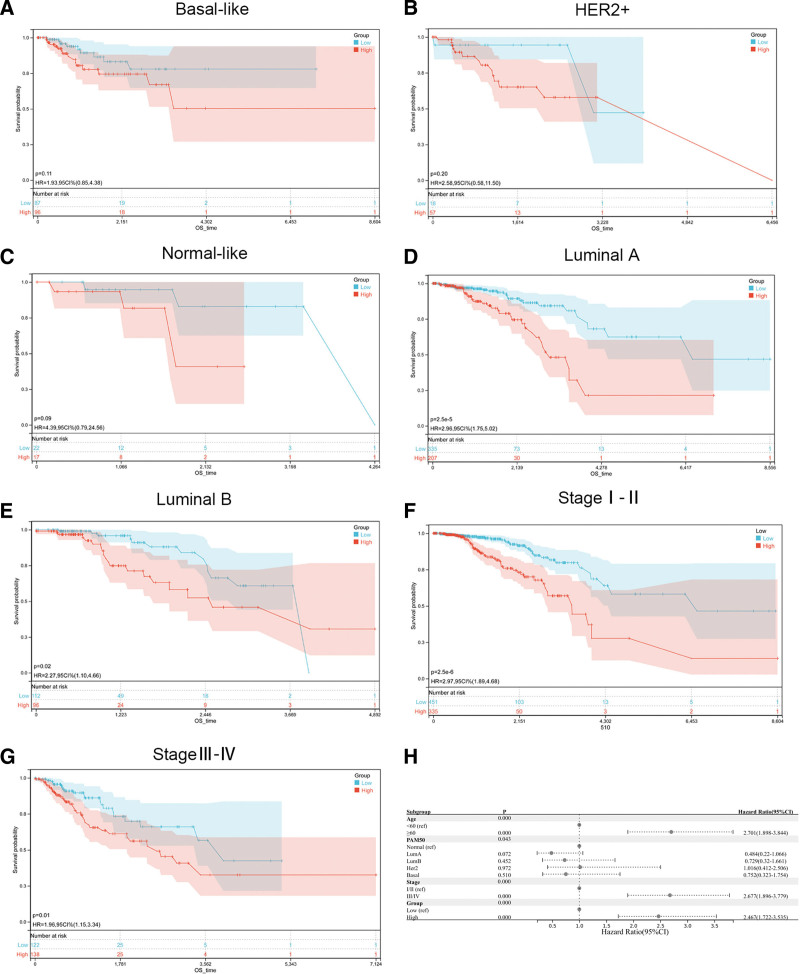
Subgroup analysis of molecular types and different stages with the GRS formula. (A) KM survival curve of the patients in the high-GRS and low-GRS groups for OS in basal-like BRCA patients. (B) KM survival curve of the patients in the high-GRS and low-GRS groups for OS in HER2 + BRCA patients. (C) KM survival curve of the patients in the high-GRS and low-GRS groups for OS in normal-like BRCA patients. (D) KM survival curve of the patients in the high-GRS and low-GRS groups for OS in Luminal A BRCA patients. (E) KM survival curve of the patients in the high-GRS and low-GRS groups for OS in Luminal B BRCA patients. (F) KM survival curve of the patients in the high-GRS and low-GRS groups for OS in stage I-II BRCA patients. (G) KM survival curve of the patients in the high-GRS and low-GRS groups for OS in stage III–VI BRCA patients. (H) Forest plot of subgroup analysis. BRCA = breast invasive carcinoma, GRS, Golgi apparatus gene-related risk score, KM = Kaplan–Meier, OS = overall survival.

The molecular subtypes and clinical stages of BRCA patients are crucial for the treatment of BC. Therefore, the GRS was used to perform molecular subtype and staging subgroup-based analyses of BRCA patients from the TCGA cohort. The formula was able to accurately group patients with luminal A and luminal B breast cancer according to prognosis (favorable or poor) but was not suitable for patients with basal-like, HER2+, and normal-like breast cancer (Fig. 5A–E). In addition, the GRS model was applicable for both early and advanced BC patients (Fig. 5F, G). The forest plots also showed that the GRS was an independent predictor of BC prognosis (Fig. 5H). We performed multivariate Cox regression analysis for subtypes with significant differences (Supplementary Table S2, Supplemental Digital Content, http://links.lww.com/MD/N356). The results showed that the GRS was still an independent prognostic factor in the luminal A and luminal B subgroups.

### 3.9. GRS groups and metabolism-related genes

**Figure 6.**
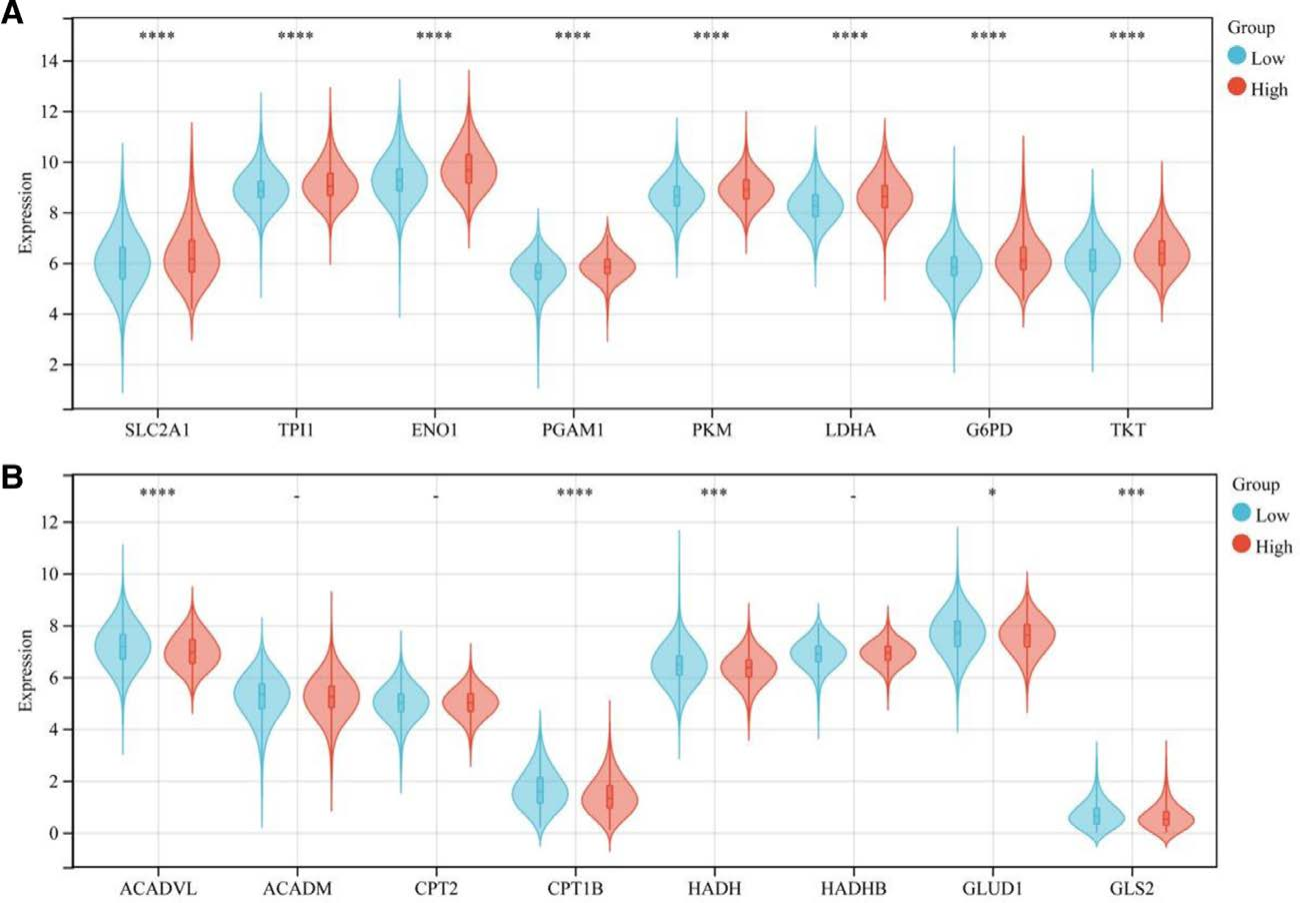
Correlation analysis between the GRS groups and metabolism-related genes. (A) Correlation analysis between the GRS groups and genes related to glycolysis and pentose phosphate pathway. (B) Correlation analysis between the GRS groups and genes related to fatty acid oxidation and glutamine metabolism. The statistical difference was compared through the Student *t* test. *P < .05; **P < .01; ***P < .001; ****P < .0001. GRS, Golgi apparatus gene-related risk score.

We assessed the expression differences in the expression of genes related to 4 metabolic pathways, namely, glycolysis, the pentose phosphate pathway, and fatty acid oxidation (FAO), and glutamine metabolism, between the high- and low-GRS groups (Fig. 6A, B). The results showed that the expression of genes related to glycolysis and the pentose phosphate pathway in the high-GRS group was greater than that in the low-GRS group, while the expression of genes involved in FAO and glutamine catabolism in the high-GRS group was lower than that in the low-GRS group.

### 3.10. Establishment of the GRS-related nomogram

**Figure 7.**
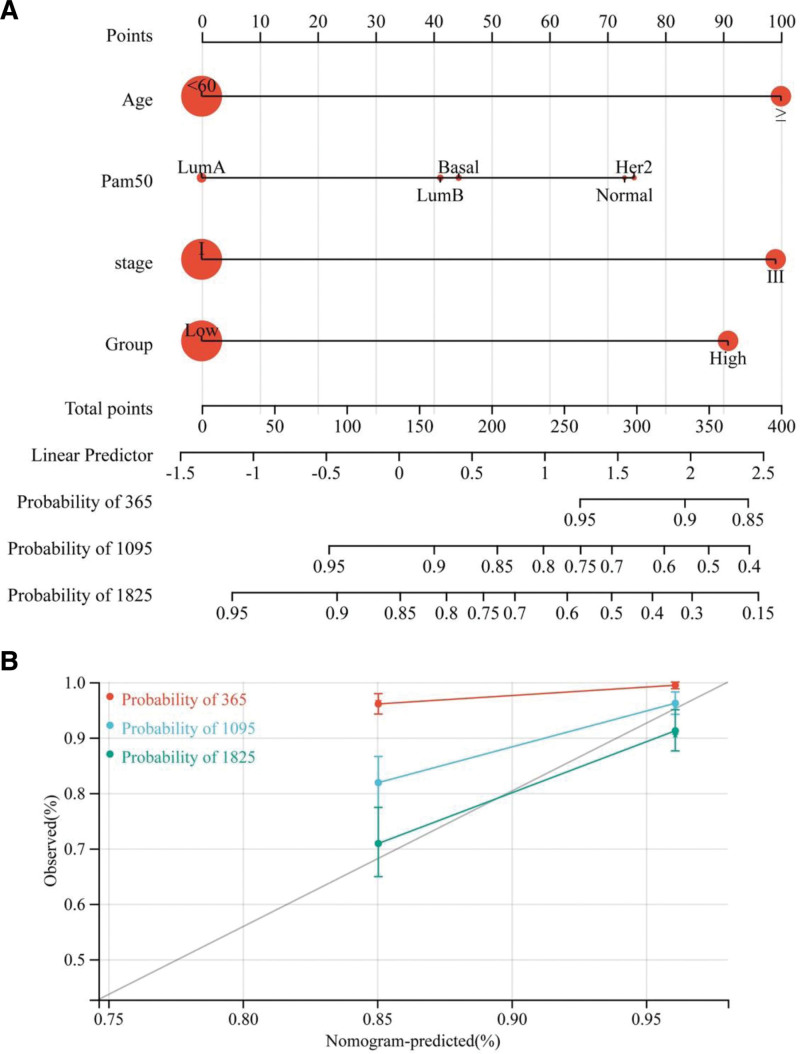
The construction of GRS-related prognostic nomogram. (A) The prognostic nomogram to predict the OS of BRCA patients. Clinical features were scored and added up to assess patient outcomes. (B) The calibration curves for predicting patient survival at 1-, 3-, and 5-yr OS. BRCA = breast invasive carcinoma, GRS, Golgi apparatus gene-related risk score, OS = overall survival.

Age, molecular subtype, stage, and GRS group were used as parameters to establish the nomogram, and all of the risk factors were used to calculate the nomogram score. The final score was compared with the prognostic patient calibration curve for survival (Fig. 7A, B). This nomogram facilitated the correlation of GRS parameters to clinical baseline data.

## 4. Discussion

BC, which has the highest incidence rate and accounts for the most cancer-related deaths in women, is an important disease endangering women’s health.^[1]^ At present, molecular typing, such as endocrine therapy and targeted therapy, is mainly used to guide the individualized treatment of BC patients. This classification is not directly related to patient prognosis. Therefore, a new formula, the GRS, was developed and verified to independently predict the prognosis of BRCA patients in this study to accurately evaluate the prognosis of BC patients and guide clinical treatment.

Of the 8 genes that make up the GRS formula, FEZ1 is a tumor suppressor gene in breast cancer associated with microtubule components, and it can inhibit cancer cell growth by regulating mitosis in breast cancer.^[22]^ The inhibition mechanism in breast cancer may be related to the downregulation of the promoter.^[23]^ The SI gene encodes a protein encoding the sucrase-isomaltase protein that is expressed in the intestinal brush margin, which plays an important role in the carbohydrate absorption function of the intestinal epithelium. APOA5 is a member of the APOA1/C3/A4/A5 gene cluster that encodes apolipoprotein A5, which can regulate the activity of lipoprotein lipase. APOA1/C3/A4/A5 is associated with a higher incidence of intracavity breast cancer in young East Asian women.^[24]^ ECE2 encodes the protein endothelin converting enzyme 2, which is associated with the aggressiveness of breast cancer.^[25]^ WNT7B is a member of the WNT gene family. In breast cancer, the Wnt/β-catenin signaling pathway is a classic cancer-related pathway,^[26]^ and the upregulation of WNT7B is associated with a poor breast cancer prognosis.^[27]^ IGFBP6 is a specific receptor for IGF-II that can inhibit the growth of malignant tumors overexpressing IGF-II, such as rhabdomyosarcoma and neuroblastoma.^[28,29]^ DUX4 is associated with glyphosate-induced gene demethylation in luminal B breast cancer, leading to cancer progression.^[30]^ The NLGN1 gene encodes a member of the neuronal cell surface protein family involved in regulating the APC/β-catenin pathway to promote colorectal cancer progression.^[31]^

We conducted functional analysis of the abnormal expression of GA-related genes in malignant breast tumors and found that they were mainly related to the biosynthesis of glycosphingolipids and glycosaminoglycans and to glycoprotein metabolism. Tumor-associated glycosphingolipids have been used as immunotherapeutic targets and diagnostic markers for cancers.^[32]^ In recent years, many studies have shown that, as tumor markers, glycosphingolipids can regulate lipid raft and membrane microdomain signaling pathways.^[33,34]^ Glycoproteins are the products of protein glycosylation and are related to the metastasis and invasion of BC.^[35]^ Analysis of glycosylation changes can be used to identify relevant BC biomarkers.^[36,37]^

The GRS formula based on 8 GA-related genes was used to predict the prognosis of BC patients, and the AUCs of the risk score for predicting 1-, 3-, and 5-year OS were 0.75, 0.75, and 0.75, respectively. The OS of the low-GRS group was significantly greater than that of the high-GRS group. The GRS also showed good predictive power in the METABRIC dataset.

TP53 and CDH1 mutations were more common in the high-GRS subgroup than in the low-GRS subgroup. As the most frequently mutated gene in BC, TP53 encodes the p53 protein, which acts as a transcription factor that controls cell cycle initiation.^[38,39]^ TP53 is a tumor suppressor gene, but when TP53 is mutated, it loses its regulatory role in cell growth, apoptosis, and DNA repair and becomes an oncogene.^[40]^ Therefore, TP53 has been regarded as a potential target for cancer drug treatment, and these targeted drugs are mainly used to transform the P53 phenotype of cancer cells from mutant to wild-type.^[41–44]^ The CDH1 gene encodes E-cadherin, a member of the cadherin family. The CDH1 gene is involved in regulating cell adhesion, migration, and epithelial cell proliferation, and its loss of function makes it easier for cells to invade and metastasize.^[45,46]^ Mutations in CDH1 are closely related to ovarian cancer, thyroid cancer, colorectal cancer, BC, and gastric cancer.^[47–51]^

We also explored the correlation between immune cell marker expression and the risk score in addition to the expression levels of markers of activated M0 macrophages and dendritic cells, those of markers of activated NK cells, CD8 + T cells, naive B cells, monocytes, and resting mast cells were inversely correlated with the GRS. That is, the high-GRS group had higher immune cell infiltration than the low-GRS group, and the immune response was also weaker. Patients in the low-GRS group responded better to immunotherapy.

In addition, we also found that BC patients with higher GRSs had increased glucose metabolism and decreased FAO and glutamine metabolism. Previous studies have shown that glucose-dependent BC has a worse prognosis than FAO- and glutamine-dependent BC, which is consistent with our conclusion.^[52]^

To combine the GRS formula with clinical features to better guide the clinical treatment of BC patients, we performed subgroup analysis of molecular subtypes and clinical stages. The results showed that the GRS formula was effective in predicting the prognosis of luminal BC but not that of patients with basal-like, HER2+, or normal-like BC. This finding suggested that the accuracy of the GRS model in differentiating the prognosis of BC patients is better for ER + patients. Considering the association of the constituent genes of the GRS formula with luminal breast cancer, such as APOA5 and DUX4, the limitations of its application in non-luminal breast cancer can be taken into account. The application of GRS model in different clinical stages is relatively comprehensive. The GRS can distinguish the prognosis of patients with early or advanced BC well.

This study has several limitations. First, the GRS formula is not applicable to all molecular types of BC, and the prognostic accuracy for ER patients is not high. Second, due to the lack of information on chemotherapy, endocrine therapy, and immunotherapy for BRCA patients in the TCGA database, we did not combine the formula with clinical treatment data to guide precise clinical treatment. Finally, due to the limitations of these conditions, we have not conducted in-depth research on the underlying mechanism. In the future, we will carry out further research and explore the underlying mechanisms in vivo and in vitro.

## 5. Conclusion

In this study, GA-related genes were used to construct a prediction model for BC patients that has high accuracy in predicting prognosis. The mutations associated with the GRS were mainly mutations in the TP53 and CDH1 genes. Interestingly, the GRS was closely related to glucose metabolism in terms of both gene expression and functional enrichment. Therefore, the role of GRS-related genes in glucose metabolism is worthy of further study. In addition, we constructed a nomogram suitable for clinical prognostic classification to facilitate clinical application of the GRS.

## Author contributions

**Data curation:** Hang Lu, Wenge Li.

**Formal analysis:** Hang Lu, Xin Yu.

**Writing—original draft:** Hang Lu, Wenge Li.

**Methodology:** Xin Yu.

**Software:** Xin Yu.

**Validation:** Xin Yu.

**Resources:** Wenge Li.

**Writing—review & editing:** Yimin Zhang, Shengrong Sun.

## Supplementary Material


