# Supplementary material for: Prognosis and metabolism with a Golgi apparatus-related genes-based formula in breast cancer
Source: Medicine (Baltimore). 2024 Aug 16;103(33):e39177. doi: 10.1097/MD.0000000000039177 (PMC11332736; doi:10.1097/MD.0000000000039177)
Supplement: Supplementary file 1 [file medi-103-e39177-s001.docx]

**Supplementary Figure S1** Univariate Cox regression analysis.

**
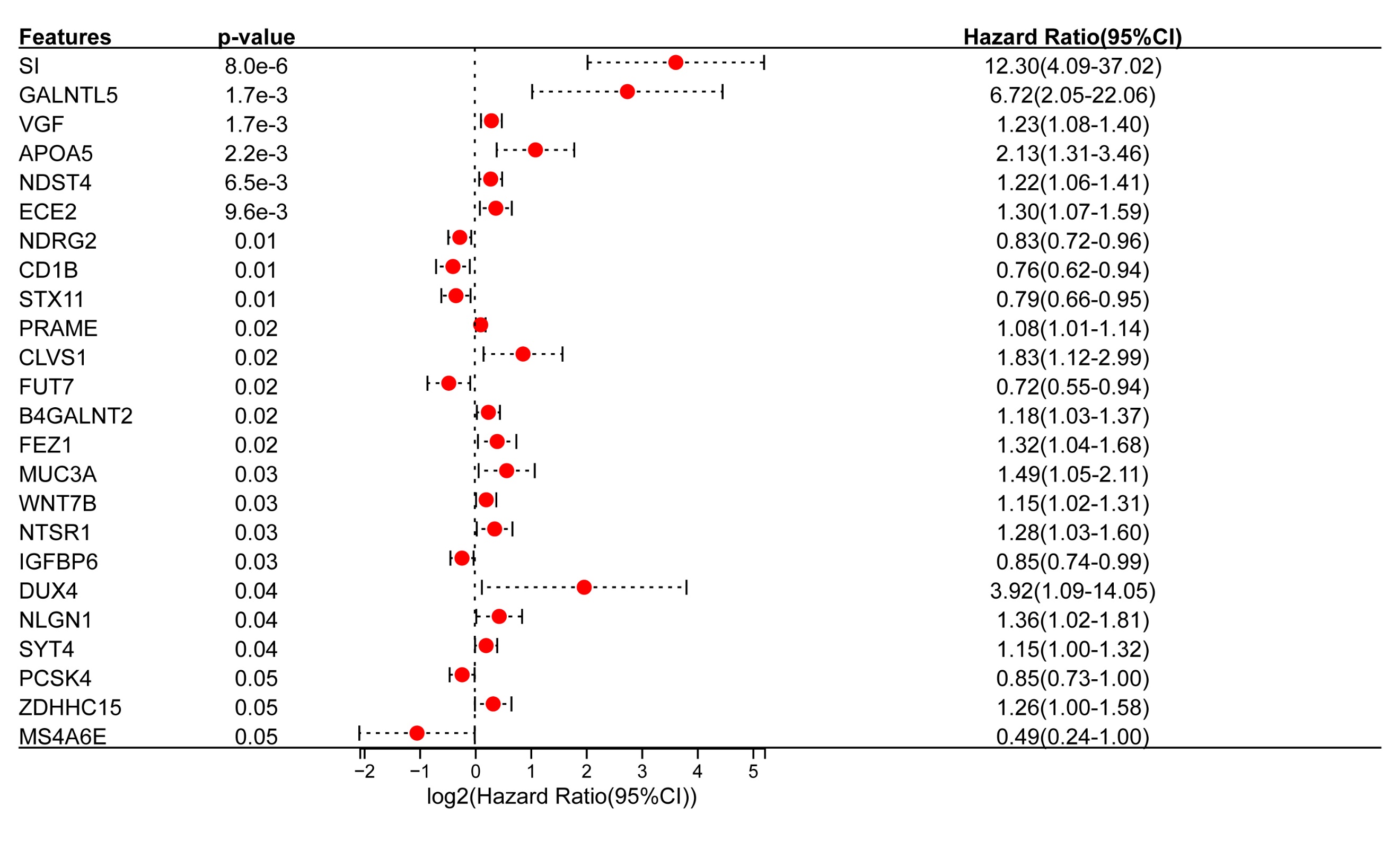
**

**Supplementary Figure S2** Multivariate Cox regression analysis.

**
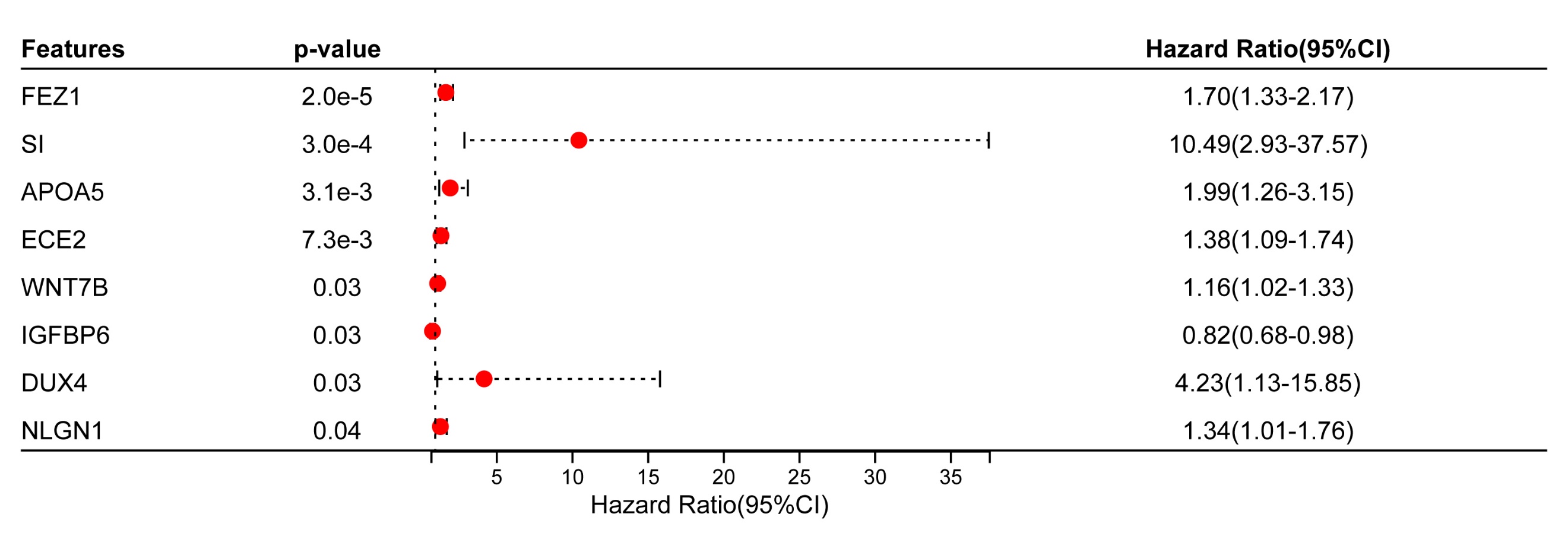
**

**Supplementary Figure S3 The DEGs between the high- and low-GRS groups.**

**
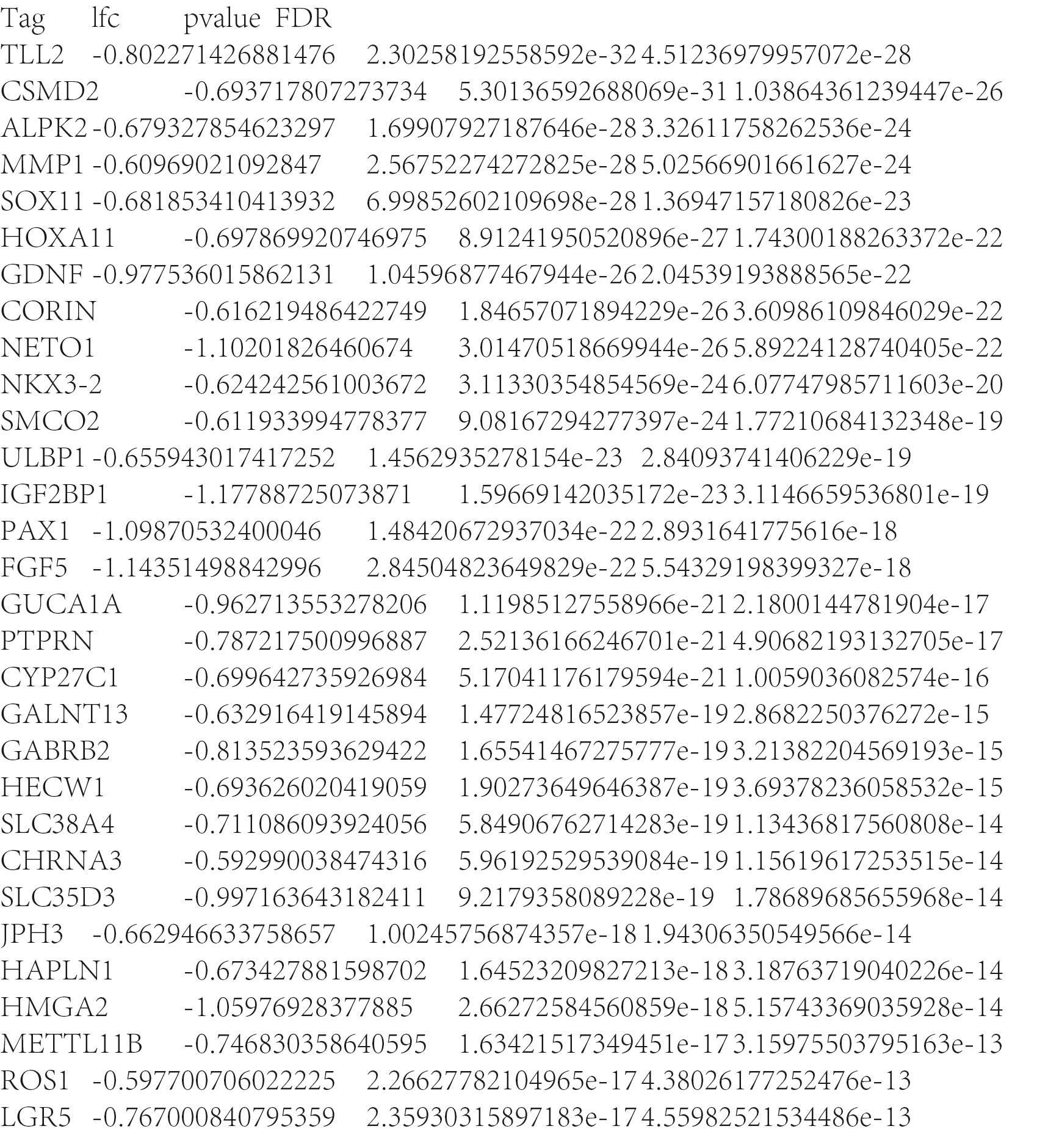
**

**Supplementary Figure S4** Functional analysis and mutation landscape in the high GRS and low GRS groups. **(A)** KEGG pathways between the high GRS and low GRS groups in TCGA-BRCA cohort. **(B)** Biological Process of GO enrichment between the high GRS and low GRS groups in TCGA-BRCA cohort. **(C)** Cellular Component of GO enrichment between the high GRS and low GRS groups in TCGA-BRCA cohort. **(D)** Molecular Function of GO enrichment between the high GRS and low GRS groups in TCGA-BRCA cohort. **(E)** Comparison of the mutation landscape between groups with high and low GRS. GRS, Golgi apparatus gene-related risk score; KEGG, Kyoto Encyclopedia of Genes and Genomes; GO, Gene Ontology.

**
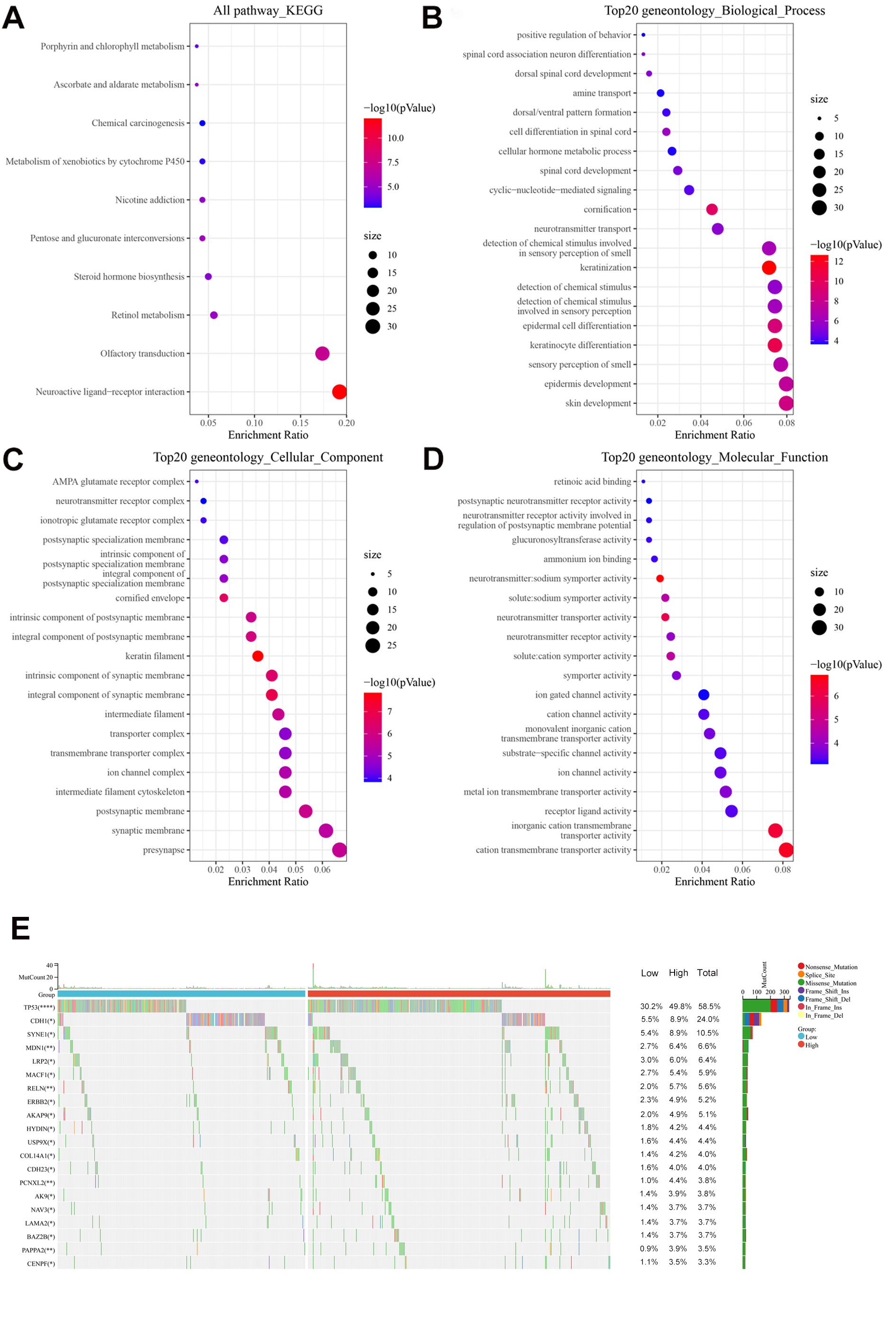
**

**Supplementary Figure S5** Correlation analysis of relative expression level of immune cells and GRS.

**
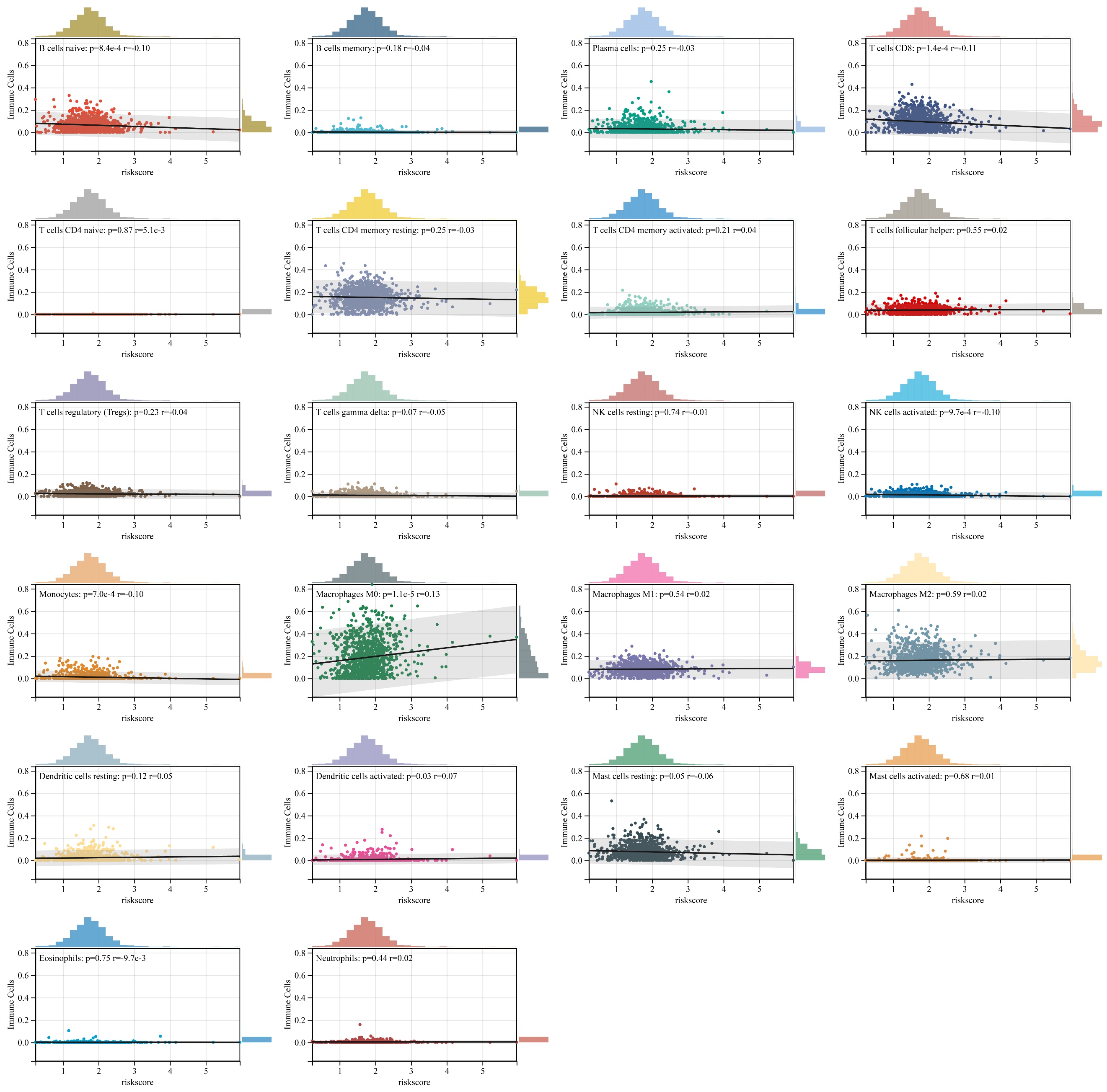
**

**Supplementary Table S1** 256 up- and down-regulated genes for DEGs analyses.

| Tag | lfc | pvalue |
| --- | --- | --- |
| MYOC | -3.61561 | 1.51E-32 |
| CA4 | -3.08407 | 1.62E-64 |
| HS3ST4 | -2.68678 | 6.85E-31 |
| LALBA | -2.61595 | 5.71E-08 |
| B3GALT1 | -2.49586 | 4.77E-32 |
| IGFBP1 | -2.32796 | 4.90E-14 |
| GOLGA8G | -2.32534 | 1.38E-06 |
| GOLGA8M | -2.15669 | 4.45E-26 |
| CAV3 | -2.00497 | 1.13E-07 |
| CRHBP | -1.98077 | 7.11E-32 |
| SLC2A4 | -1.94512 | 6.96E-45 |
| NLGN1 | -1.9347 | 8.20E-40 |
| GKN1 | -1.83262 | 1.60E-06 |
| CHST9 | -1.79961 | 2.85E-29 |
| DRD2 | -1.63015 | 1.84E-27 |
| TENM1 | -1.61852 | 2.15E-33 |
| MGAT4C | -1.61456 | 0.003767322 |
| CABP1 | -1.58906 | 1.01E-34 |
| ABCB11 | -1.56249 | 2.04E-08 |
| AVPR2 | -1.52544 | 6.33E-62 |
| GOLGA8T | -1.48381 | 5.96E-14 |
| ALB | -1.47598 | 5.69E-19 |
| MUC7 | -1.47384 | 2.38E-06 |
| PRCD | -1.40829 | 2.97E-46 |
| HPD | -1.40605 | 3.16E-14 |
| FAT2 | -1.40076 | 3.23E-39 |
| PDE3B | -1.39912 | 6.54E-26 |
| ABCA6 | -1.39823 | 6.55E-55 |
| GALNT8 | -1.38585 | 1.80E-30 |
| GOLGA8K | -1.3654 | 8.05E-16 |
| MS4A6E | -1.34322 | 0.001450169 |
| NMNAT2 | -1.3421 | 6.38E-45 |
| NCAM1 | -1.33382 | 2.00E-42 |
| GOLGA8Q | -1.30031 | 3.01E-11 |
| ADRB2 | -1.29002 | 6.85E-78 |
| B3GAT1 | -1.28521 | 1.07E-12 |
| CALN1 | -1.23313 | 2.25E-14 |
| RIC3 | -1.21937 | 4.18E-33 |
| PDE2A | -1.20411 | 2.58E-73 |
| CLVS1 | -1.19687 | 1.13E-16 |
| ST8SIA2 | -1.18886 | 3.78E-13 |
| TENM2 | -1.18849 | 9.11E-24 |
| MME | -1.18294 | 5.87E-123 |
| HSPB6 | -1.15525 | 6.21E-59 |
| MUC15 | -1.13092 | 6.84E-24 |
| CD36 | -1.12455 | 2.76E-65 |
| CUBN | -1.10314 | 7.05E-38 |
| GAL3ST1 | -1.10274 | 1.10E-17 |
| GOLGA8R | -1.09934 | 1.31E-21 |
| GALNT15 | -1.0941 | 1.47E-52 |
| GOLGA8O | -1.08939 | 3.50E-07 |
| F10 | -1.08624 | 1.83E-46 |
| MUC3A | -1.07092 | 3.91E-20 |
| GOLGA8H | -1.06809 | 9.15E-23 |
| A4GNT | -1.06351 | 2.08E-08 |
| ST6GALNAC3 | -1.05907 | 8.23E-43 |
| ST6GALNAC1 | -1.05399 | 1.02E-42 |
| CLIC5 | -1.03303 | 1.92E-54 |
| GOLGA8N | -1.0224 | 6.34E-32 |
| GOLGA8S | -1.01977 | 5.82E-10 |
| SEMA6D | -1.00162 | 4.22E-52 |
| YIPF7 | -0.99437 | 0.031336439 |
| LGR6 | -0.97305 | 4.26E-22 |
| OGN | -0.95222 | 3.62E-71 |
| FOLR1 | -0.94697 | 7.16E-23 |
| SYNDIG1L | -0.94424 | 1.07E-09 |
| MPL | -0.9397 | 4.55E-31 |
| MS4A4E | -0.92856 | 9.80E-22 |
| EGFR | -0.90308 | 4.11E-132 |
| PLA2G5 | -0.90081 | 8.60E-29 |
| GPC3 | -0.89663 | 2.74E-88 |
| SLC35G2 | -0.88639 | 8.21E-42 |
| PTCH1 | -0.8787 | 7.55E-54 |
| PLA2G4A | -0.86333 | 6.10E-90 |
| MUC4 | -0.858 | 1.81E-05 |
| DEFB1 | -0.85212 | 2.12E-39 |
| MGAT3 | -0.84899 | 9.80E-45 |
| KLK11 | -0.84785 | 3.37E-14 |
| AOC3 | -0.84483 | 5.80E-48 |
| ALX1 | -0.84061 | 3.47E-06 |
| KCNA5 | -0.83578 | 1.36E-13 |
| APBA1 | -0.81969 | 1.33E-42 |
| CRYAB | -0.8171 | 1.70E-96 |
| ZDHHC15 | -0.81412 | 1.64E-44 |
| FGF7 | -0.81247 | 9.62E-52 |
| MUCL1 | -0.80166 | 2.79E-18 |
| CAV2 | -0.80017 | 2.10E-71 |
| ABO | -0.79965 | 1.98E-34 |
| FUT6 | -0.79708 | 1.55E-06 |
| PLEKHM3 | -0.79616 | 1.19E-31 |
| PLAGL1 | -0.79529 | 1.09E-72 |
| NLRP5 | -0.78943 | 3.59E-05 |
| IGFBP6 | -0.77189 | 3.52E-60 |
| GCNT4 | -0.76779 | 2.17E-31 |
| B3GALT2 | -0.76516 | 1.31E-14 |
| PCSK5 | -0.76278 | 9.97E-37 |
| HEPACAM2 | -0.76009 | 4.58E-12 |
| P3H2 | -0.75937 | 4.24E-48 |
| ARL5C | -0.75848 | 3.39E-06 |
| AQP2 | -0.75705 | 0.000320759 |
| ST8SIA1 | -0.75023 | 5.06E-22 |
| GAL3ST3 | -0.74967 | 0.001595911 |
| ATP8B4 | -0.73664 | 2.41E-26 |
| GPC5 | -0.71992 | 4.87E-09 |
| SGSM1 | -0.71543 | 1.41E-21 |
| PKHD1 | -0.71151 | 0.001469483 |
| SOD3 | -0.70983 | 1.12E-58 |
| KCNJ2 | -0.70572 | 2.11E-37 |
| TPPP | -0.7038 | 1.51E-54 |
| FEZ1 | -0.69861 | 5.56E-47 |
| WNT6 | -0.69777 | 5.17E-13 |
| PKDCC | -0.68363 | 8.65E-29 |
| PRKD1 | -0.681 | 8.96E-75 |
| ABCA5 | -0.67746 | 2.70E-58 |
| NCALD | -0.65701 | 2.96E-43 |
| ITM2A | -0.65609 | 9.61E-145 |
| STX11 | -0.65576 | 1.05E-26 |
| SYNE1 | -0.6533 | 7.32E-56 |
| DSEL | -0.6532 | 2.24E-47 |
| MMP24 | -0.64802 | 5.00E-21 |
| CAV1 | -0.64569 | 6.76E-73 |
| CLCN4 | -0.63892 | 1.46E-35 |
| B4GALT6 | -0.63655 | 7.62E-42 |
| MUC6 | -0.62903 | 1.74E-06 |
| SEC16B | -0.62854 | 1.17E-12 |
| PDE9A | -0.62584 | 3.67E-47 |
| PROS1 | -0.6254 | 8.81E-92 |
| B3GNT5 | -0.62297 | 5.32E-32 |
| GIMAP1 | -0.61779 | 1.96E-58 |
| F8 | -0.61333 | 8.18E-54 |
| STEAP4 | -0.61003 | 1.47E-69 |
| GIMAP8 | -0.60734 | 1.01E-60 |
| GALNT12 | -0.60291 | 1.14E-25 |
| MALL | -0.59698 | 1.27E-27 |
| NDRG2 | -0.59681 | 8.29E-70 |
| PRELP | -0.59679 | 2.21E-50 |
| PDGFD | -0.58777 | 1.14E-74 |
| GPER1 | -0.58695 | 3.29E-29 |
| F5 | 0.58556 | 1.80E-09 |
| ATP8B3 | 0.591972 | 1.74E-24 |
| GAL | 0.593261 | 1.39E-14 |
| CSPG5 | 0.593308 | 4.78E-22 |
| CD1B | 0.593886 | 4.12E-07 |
| CAMK1G | 0.606755 | 1.13E-08 |
| GDF15 | 0.614024 | 2.92E-25 |
| B3GNT4 | 0.619466 | 1.05E-18 |
| NDST3 | 0.621564 | 4.93E-06 |
| FUT2 | 0.632524 | 4.30E-21 |
| CHST1 | 0.636182 | 7.24E-23 |
| PCSK4 | 0.638655 | 2.14E-22 |
| NOD2 | 0.64197 | 2.60E-27 |
| ABCA12 | 0.647932 | 2.79E-08 |
| MSLN | 0.665362 | 9.09E-08 |
| MUC5B | 0.668128 | 8.76E-14 |
| NAA11 | 0.670766 | 0.027597667 |
| WNT7B | 0.674829 | 7.60E-22 |
| APLP1 | 0.68608 | 1.46E-39 |
| TMEM132A | 0.68967 | 4.75E-35 |
| MALRD1 | 0.69029 | 7.40E-10 |
| CLEC18A | 0.690852 | 1.39E-06 |
| DEFA4 | 0.694203 | 0.041059986 |
| GPR143 | 0.697344 | 3.28E-25 |
| B4GALNT4 | 0.709194 | 1.96E-25 |
| CLEC18B | 0.711424 | 0.000110681 |
| SH3GL2 | 0.711734 | 7.13E-12 |
| ADAM19 | 0.719841 | 2.36E-47 |
| FGFR3 | 0.726335 | 4.64E-31 |
| ST8SIA5 | 0.741164 | 1.88E-07 |
| JAKMIP2 | 0.751961 | 7.14E-17 |
| UMOD | 0.817046 | 5.73E-10 |
| GPC2 | 0.828474 | 2.65E-24 |
| GBP5 | 0.829669 | 2.98E-38 |
| PCSK9 | 0.838979 | 6.31E-10 |
| SYN1 | 0.84113 | 4.04E-32 |
| CLEC18C | 0.84837 | 0.000819438 |
| HS3ST6 | 0.871375 | 6.95E-05 |
| B3GNT6 | 0.871439 | 0.003356446 |
| PCSK1N | 0.872903 | 4.05E-16 |
| ECE2 | 0.874083 | 1.37E-53 |
| RHO | 0.882773 | 2.30E-09 |
| MAPK15 | 0.883958 | 3.42E-33 |
| AGRP | 0.887516 | 3.55E-15 |
| ATG9B | 0.911837 | 7.49E-58 |
| MGAT5B | 0.938665 | 1.20E-33 |
| GDNF | 0.942109 | 5.45E-05 |
| COLEC10 | 0.952159 | 2.77E-24 |
| WNT7A | 0.955376 | 1.02E-05 |
| B4GALNT2 | 0.985676 | 5.01E-07 |
| CIT | 0.987989 | 9.27E-60 |
| FUT5 | 1.030508 | 1.98E-05 |
| CDH15 | 1.04964 | 1.47E-05 |
| PCSK1 | 1.053922 | 4.47E-16 |
| CREG2 | 1.068645 | 1.15E-23 |
| GCNT3 | 1.093655 | 2.74E-10 |
| MUC21 | 1.135939 | 0.02159364 |
| DYNAP | 1.169008 | 0.000176695 |
| F7 | 1.17524 | 1.95E-36 |
| PROZ | 1.198179 | 3.55E-32 |
| CABP7 | 1.252284 | 3.33E-37 |
| GALNTL5 | 1.266225 | 0.010487618 |
| HS3ST3A1 | 1.273528 | 4.75E-60 |
| RAB26 | 1.312929 | 5.52E-65 |
| GAD2 | 1.315274 | 0.000377429 |
| SYT4 | 1.32883 | 0.000783703 |
| CHST6 | 1.338097 | 6.30E-47 |
| MUC13 | 1.395681 | 3.74E-12 |
| SLC35D3 | 1.401247 | 5.72E-10 |
| PTPRN | 1.403286 | 3.38E-09 |
| CREB3L3 | 1.416811 | 1.62E-11 |
| FGF23 | 1.443641 | 0.042943796 |
| KIF20A | 1.44844 | 7.83E-67 |
| NCAN | 1.449834 | 0.001130131 |
| AHSG | 1.450402 | 0.000595558 |
| HS3ST5 | 1.496776 | 9.63E-08 |
| FUT7 | 1.502958 | 5.26E-35 |
| AVPR1B | 1.536337 | 5.96E-11 |
| ENPP7 | 1.550176 | 1.23E-15 |
| SHH | 1.554366 | 1.38E-14 |
| APOA5 | 1.557115 | 1.45E-09 |
| HTR5A | 1.608056 | 0.014043104 |
| NTSR1 | 1.612826 | 1.34E-09 |
| POLQ | 1.621248 | 7.95E-75 |
| CLSPN | 1.632491 | 2.13E-81 |
| MGAT4D | 1.697341 | 7.32E-07 |
| AMELX | 1.73401 | 0.000495803 |
| ESCO2 | 1.738905 | 2.97E-72 |
| SLC30A8 | 1.757842 | 1.61E-27 |
| ZG16 | 1.819315 | 9.30E-17 |
| F2 | 1.826719 | 1.38E-12 |
| ATP1A3 | 1.827279 | 4.21E-66 |
| PROC | 1.829236 | 1.19E-87 |
| TRPC7 | 1.896649 | 2.31E-07 |
| ACAN | 1.961048 | 1.54E-64 |
| MMP11 | 1.972827 | 7.43E-157 |
| CNTNAP2 | 1.984476 | 2.30E-54 |
| GFY | 2.033797 | 2.14E-10 |
| MUC5AC | 2.058846 | 2.42E-05 |
| PKMYT1 | 2.086754 | 2.72E-95 |
| PRAME | 2.129125 | 5.62E-42 |
| NDST4 | 2.280699 | 2.21E-10 |
| KCNJ6 | 2.296203 | 2.95E-32 |
| SLC24A5 | 2.300441 | 4.56E-12 |
| TXNDC8 | 2.33971 | 1.26E-08 |
| SI | 2.365847 | 0.002840612 |
| INS | 2.496688 | 0.00273782 |
| DEFB4A | 2.694207 | 0.000284581 |
| ZDHHC22 | 2.701916 | 1.22E-17 |
| GAL3ST2 | 2.724118 | 8.54E-59 |
| SPATA16 | 2.814617 | 1.78E-07 |
| SLC18A3 | 2.965237 | 4.39E-05 |
| VGF | 2.994103 | 1.19E-60 |
| OLFM3 | 3.08282 | 2.17E-09 |
| DUX4 | 3.200096 | 0.000683294 |
| CGA | 3.591628 | 8.45E-51 |
| TAS2R16 | Inf | 0.032604515 |
| DEFB103A | Inf | 0.049038102 |

**Supplementary Table S2** multivariate COX regression analysis for Luminal A and Luminal B subgroups.

|  | P value | Exp(B) | 95.0%CI | |
| --- | --- | --- | --- | --- |
|  |  |  | Upper limit | Lower limit |
| Age (≥60 *v.s.* <60) | **0.000** | 2.998 | 1.709 | 5.257 |
| Stage (Ⅲ/Ⅳ *v.s.* Ⅰ/Ⅱ) | **0.017** | 1.924 | 1.122 | 3.300 |
| Group (High *v.s.* Low) | **0.000** | 2.996 | 1.744 | 5.147 |

Pam50 = LumA

|  | P value | Exp(B) | 95.0%CI | |
| --- | --- | --- | --- | --- |
|  |  |  | Upper limit | Lower limit |
| Age (≥60 *v.s.* <60) | **0.023** | 2.371 | 1.128 | 4.982 |
| Stage (Ⅲ/Ⅳ *v.s.* Ⅰ/Ⅱ) | **0.014** | 2.452 | 1.197 | 5.026 |
| Group (High *v.s.* Low) | **0.013** | 2.523 | 1.220 | 5.216 |

Pam50 = LumB
